# Mal de Debarquement Syndrome: A Case Presentation of a Vestibular Enigma

**DOI:** 10.7759/cureus.65787

**Published:** 2024-07-30

**Authors:** Deepika Nagliya, Sonia Daryanani

**Affiliations:** 1 College of Osteopathic Medicine, Nova Southeastern University Dr. Kiran C. Patel College of Osteopathic Medicine, Fort Lauderdale, USA; 2 Allergy and Immunology, Nova Southeastern University Dr. Kiran C. Patel College of Osteopathic Medicine, Fort Lauderdale, USA

**Keywords:** vestibulo-ocular reflex, vertigo, vestibular adaptation, vestibular disorder, mal de debarquement

## Abstract

Mal de Debarquement (MdD) is a rare vestibular disorder characterized by a rocking or swaying sensation following passive motion exposure, such as boat or airplane travel. The etiology and pathogenesis are unknown. Due to a lack of clinical awareness and research, it is underrecognized and misdiagnosed. We present a case of a 37-year-old male with classic MdD symptoms following an amusement park boat ride. Along with normal evaluations, including neurological and vestibular tests, the patient experienced relief with clonazepam, aligned with current management strategies. MdD's impact extends beyond physical symptoms, with studies showing significant economic and psychological burdens associated with the condition. Therefore, it is important to recognize MdD as a missed diagnosis that can potentially result in prolonged and debilitating symptoms that may require management with selective serotonin reuptake inhibitors (SSRIs), transcranial magnetic stimulation, or vestibular rehabilitation.

## Introduction

Mal de Debarquement (MdD) is a rare disorder of the vestibular system often triggered by exposure to passive motion such as boat or airplane travel [1]. The pathogenesis of MdD remains poorly understood. MdD is a clinical diagnosis. The characteristic symptom is a persistent rocking or swaying sensation, typically occurring after exposure to motion, without associated nausea, vomiting, or a sensation of spinning [1,2]. Hearing is usually preserved, and the neurologic examination of these patients is notably benign [2]. Testing is useful only in helping to exclude other disorders that might present with features like MdD. There is a significant delay between the onset of symptoms and an accurate diagnosis due to a lack of awareness about the disorder. This case report aims to raise awareness and improve understanding and management of MdD in the medical community, as the condition is often underrecognized, and increased awareness can significantly improve the quality of life of patients and reduce financial costs associated with their care.

## Case presentation

**Figure 1:**
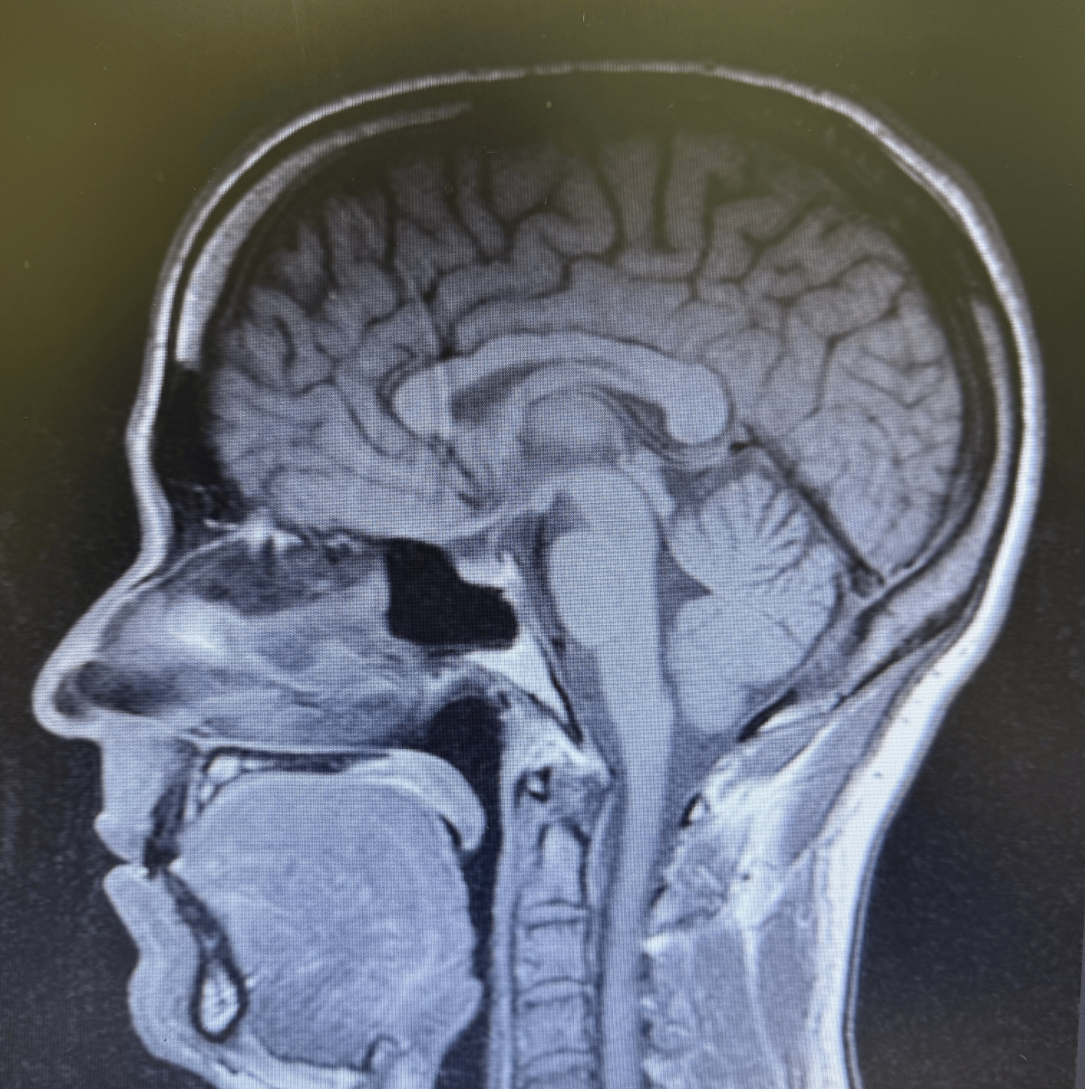
Magnetic resonance imaging (MRI) of the brain sagittal view showing normal findings

We present a case of a 37-year-old male with a past medical history significant for infrequent episodes of motion sickness, who presented to the outpatient clinic with a complaint of dizziness for a month before presentation. He described it as a “rocking on a boat” sensation. Episodes lasted 30-60 minutes, and he reported feeling "off" for the rest of the day. The symptoms began after a ride on Pirates of the Caribbean, a boat ride at Disney World. Emergency Medical Services (EMS) evaluated him shortly after the Pirates of the Caribbean ride when his symptoms started. According to the patient his vital signs, blood glucose, and electrocardiogram were normal at the time of this evaluation. The patient had three subsequent symptomatic motion sickness episodes. Over-the-counter meclizine, previously effective, provided no relief. The patient was training for a marathon but was consistently staying well-hydrated and following nutritional advice from his dietician. He denied other symptoms like blurry vision, vomiting, headache, nausea, tinnitus, and hearing loss. The patient denied any history of tobacco use, admitted to socially consuming alcohol, and having caffeine daily from one cup of coffee in the morning and a Celsius® energy drink after lunch. He had no known history of trauma, injury, ear infection, cardiac disease, or neurological disease including an aneurysm or tumor. His vital signs were temperature 97.8 F, heart rate 63 bpm, respiratory rate 16 rpm, BP 114/76 mmHg. He had a thorough clinical examination, including neurological and vestibular tests, all of which were unremarkable. Laboratory tests were normal (Table 1), and magnetic resonance imaging (MRI) of the brain revealed no evidence of intra-axial mass, extra-axial mass, hemorrhage, acute ischemia, edema, or midline shift (Figure 1). The patient was evaluated by a neurologist to help rule out other potential causes of dizziness and imbalance. Based on the history of symptoms triggered after disembarking from a boat ride, the characteristic "rocking" sensation, the clinical presentation, and unremarkable neurological and imaging findings, the patient was diagnosed with MdD. The neurologist concurred with the treatment which included clonazepam for symptomatic relief and did not recommend further workup or evaluation by otolaryngology. During his follow-up visit, the patient reported feeling immediate relief with just one clonazepam during each subsequent episode of dizziness.

**Table 1:** Laboratory results for the patient, including the complete blood count (CBC), vitamin B12, serum folate levels, and the basic metabolic panel (BMP), along with their respective normal ranges WBC: white blood cell; MCV: mean corpuscular volume; MCHC: mean corpuscular hemoglobin concentration; RDW: red cell distribution width; eGFR: estimated glomerular filtration rate; BUN: blood urea nitrogen

Test	Result	Reference range
Complete blood count (CBC)		
WBC	6.5 x 10^3^/L	3.9-10.7 x 10^3^/L
Hemoglobin	14.8 g/dL	12.0-14.7 g/dL
Hematocrit	44.10%	37.1-45.4%
MCV	91.3 fL	81.9-99.9 fL
MCHC	33.6 g/dL	30.9-33.6 g/dL
RDW	12.20%	11.5-15.0%
Platelets	200 x 10^3^/L	126-373 x 10^3^/L
Vitamin B12	585 pg/mL	200-1100 pg/mL
Folate, serum	15.9 ng/mL	>5.4 ng/mL
Basic metabolic panel (BMP)		
Sodium	139 mEq/L	135-146 mEq/L
Potassium	4.4 mEq/L	3.5-5.3 mEq/L
Chloride	103 mEq/L	98-110 mEq/L
CO_2_	28 mEq/L	20-32 mEq/L
BUN	19 mg/dL	7-25 mg/dL
Creatinine	1.22 mg/dL	0.60-1.26 mg/dL
eGFR	78 mL/min/1.73m^2^	>60 mL/min/1.73m^2^

## Discussion

MdD is considered a rare disorder with an unknown prevalence in the general population. However, transient MdD symptoms following passive motion are common, with reported rates of 59% on a ship motion simulator and up to around 72-80% after actual sea travel [3]. Hain et al. discussed that it predominantly affects middle-aged females [4], while our patient is a middle-aged male. The pathogenesis of MdD remains poorly understood, with several theories proposed to explain its mechanisms. One hypothesis suggests that it is a disorder of neuroplasticity, specifically related to vestibular-ocular reflex (VOR) maladaptation [2]. Genetic factors and reliance on the somatosensory system may also play a role, like motion sickness [2]. These theories highlight the complex interplay of neuroplasticity, genetic factors, and sensory systems in the pathogenesis of MdD. However, further research is needed to explain the exact mechanisms underlying this condition.

Van Ombergen et al. propose a set of diagnostic guidelines for healthcare professionals encountering patients with suspected MdD, aimed at facilitating timely diagnosis and potentially reducing the socio-economic impact on patients' lives [3]. These guidelines are based on a comprehensive review of literature and clinical experience. MdD is characterized by chronic rocking dizziness triggered by passive motion such as travel or virtual reality exposure. Symptoms should persist for at least one month with normal inner ear function confirmed via electronystagmography (ENG) and videonystagmography (VNG), audiological tests, and structural brain imaging. The diagnosis should not be better explained by another condition [3]. The International Classification of Vestibular Disorders by the Bárány Society provides additional criteria for diagnosing MdD, including non-spinning vertigo characterized by an oscillatory perception, such as rocking, bobbing, or swaying, present continuously or for most of the day, onset occurring within 48 hours after the end of exposure to passive motion, temporary symptom reduction with exposure to passive motion, such as driving, and symptoms persisting for more than 48 hours [5]. While these guidelines aim to provide a framework for diagnosing MdD, further research is needed to establish a consensus on MdD diagnostic criteria. Most cases are self-limited, but if symptoms persist, treatment options are limited. There is no effective treatment for MdD, but benzodiazepines can provide limited symptomatic relief; clonazepam 0.25 mg to 0.5 mg orally twice daily is most often used [4,6]. Dai et al. proposed VOR modulation as a therapeutic approach for MdD, where patients underwent head rolling at their rocking frequency while watching a rotating visual stimulus, resulting in significant symptom reduction for 70% of patients lasting at least four months, suggesting that readapting the VOR could be effective in managing MdD [7].

The patient's positive response to clonazepam aligns with current management strategies. Given the clinical nature of the diagnosis, the absence of findings on audiological and neurological examination and normal imaging further supports the MdD diagnosis. The temporal association with motion exposure and lack of other significant symptoms collectively contribute to the clinical picture. 

Understanding the economic burden and psychological impact of MdD is crucial for improving patient outcomes. Macke et al.’s study highlighted the economic burden of MdD, revealing that patients with MdD experienced poor quality of life and incurred an average cost of approximately $2,997 for diagnosis, visiting no fewer than 19 physicians on average [8]. This journey to diagnosis often involves months of suffering and numerous referrals due to a lack of awareness about MdD among healthcare professionals. Stress also plays a crucial role in MdD, with significant psychological impacts reported, including anxiety and depression. This is evidenced by Riley et al.’s study, which showed significantly higher scores on the Patient Health Questionnaire-9 (PHQ-9) depression scale and the Generalized Anxiety Disorder 7 (GAD-7) anxiety scale among MdD patients compared to the normal population [9]. This emphasizes the importance of addressing mental health aspects in MdD management, as it can significantly impact symptom severity and overall well-being.

## Conclusions

Despite its rarity, MdD’s impact on individuals can be significant due to its high economic and psychological burdens. MdD’s diagnosis is challenging due to limited awareness, standardized diagnostic criteria, and specific biomarkers. The complexity of MdD necessitates a collaborative effort that bridges the gaps between neurological, vestibular, and psychological specialists, leading to better patient care and a deeper understanding of this condition. Continued research is crucial to enhancing our understanding of MdD's pathogenesis and refining treatment options. Potential areas for future research include exploring genetic factors to understand individual susceptibility and potential targeted therapies. Additionally, developing and validating novel diagnostic techniques and evaluating new treatment modalities like transcranial magnetic stimulation could significantly improve the diagnosis and management of MdD. Raising awareness of this disorder among physicians can help with an early diagnosis, thereby facilitating appropriate and early interventions for the patient, resulting in improved outcomes.
